# Rapid accumulation of HIV-1 thymidine analogue mutations and phenotypic impact following prolonged viral failure on zidovudine-based first-line ART in sub-Saharan Africa

**DOI:** 10.1093/jac/dkw583

**Published:** 2017-02-04

**Authors:** Ruth L. Goodall, David T. Dunn, Peter Nkurunziza, Lincoln Mugarura, Theresa Pattery, Paula Munderi, Cissy Kityo, Charles Gilks, Pontiano Kaleebu, Deenan Pillay, Ravindra K. Gupta, on behalf of the DART Virology Group

**Affiliations:** 1 MRC CTU at UCL, London, UK; 2 MRC/UVRI Uganda Research Unit on AIDS, Entebbe, Uganda; 3 Joint Clinical Research Centre, Kampala, Uganda; 4 Janssen Diagnostics, a division of Janssen Pharmaceuticals NV, Beerse, Belgium; 5 School of Population Health, University of Queensland, Brisbane, Australia; 6 Africa Health Research Institute, KwaZulu Natal, South Africa; 7 Division of Infection and Immunity, University College London, London, UK

## Abstract

**Background:** Lack of viral load monitoring of ART is known to be associated with slower switch from a failing regimen and thereby higher prevalence of MDR HIV-1. Many countries have continued to use thymidine analogue drugs despite recommendations to use tenofovir in combination with a cytosine analogue and NNRTI as first-line ART. The effect of accumulated thymidine analogue mutations (TAMs) on phenotypic resistance over time has been poorly characterized in the African setting.

**Patients and methods:** A retrospective analysis of individuals with ongoing viral failure between weeks 48 and 96 in the NORA (Nevirapine OR Abacavir) study was conducted. We analysed 36 genotype pairs from weeks 48 and 96 of first-line ART (14 treated with zidovudine/lamivudine/nevirapine and 22 treated with zidovudine/lamivudine/abacavir). Phenotypic drug resistance was assessed using the Antivirogram assay (v. 2.5.01, Janssen Diagnostics).

**Results:** At 96 weeks, extensive TAMs (≥3 mutations) were present in 50% and 73% of nevirapine- and abacavir-treated patients, respectively. The mean (SE) number of TAMs accumulating between week 48 and week 96 was 1.50 (0.37) in nevirapine-treated participants and 1.82 (0.26) in abacavir-treated participants. Overall, zidovudine susceptibility of viruses was reduced between week 48 [geometric mean fold change (FC) 1.3] and week 96 (3.4, *P *=* *0.01). There was a small reduction in tenofovir susceptibility (FC 0.7 and 1.0, respectively, *P *=* *0.18).

**Conclusions:** Ongoing viral failure with zidovudine-containing first-line ART is associated with rapidly increasing drug resistance that could be mitigated with effective viral load monitoring.

## Introduction

Combination ART (cART) has led to declining mortality and HIV-1 incidence in high-prevalence settings.^1^^,^^2^ WHO recommends tenofovir/lamivudine/emtricitabine as the NRTI backbone of cART.^3^ Although countries have scaled up use of tenofovir, thymidine analogues such as stavudine or zidovudine are still in use. Virological failure occurs in 15%–35% of patients treated with thymidine analogue-containing first-line cART within the first year, with the majority harbouring resistance to NNRTI and lamivudine in areas without access to viral load monitoring.^4^^,^^5^ As tenofovir becomes available, increasing numbers of individuals are substituting thymidine analogues for tenofovir without documented viral suppression at switch, carrying risk for acquisition of drug resistance.^4^^,^^5^ There is also evidence pointing to prior undisclosed thymidine analogue-containing ART in patients presenting for first-line tenofovir-based ART in sub-Saharan Africa.^6^^,^^7^ Unsurprisingly, therefore, thymidine analogue mutations (TAMs) have been reported in patients following viral failure of tenofovir-containing first-line regimens,^8^ contributing to multidrug resistance in these individuals.^9^ As limitation of emergence and transmission of MDR HIV is a priority, understanding the accumulation and phenotypic impact of TAMs remains important.

Despite a large body of data on HIV-1 resistance mutations in cross-sectional adult treatment studies from Africa,^10^ there are few longitudinal data on accumulation of drug resistance during prolonged viral failure of first-line cART regimens, and even less information on the phenotypic impact of such accumulated mutations. We sought to address these gaps in current knowledge through a retrospective analysis of individuals with ongoing viral failure between 48 and 96 weeks in the NORA (Nevirapine OR Abacavir) sub-study of the DART (Development of AntiRetroviral Therapy in Africa) trial.

## Patients and methods

DART-NORA enrolled 600 previously untreated and asymptomatic Ugandan patients with CD4 counts of <200 cells/mm^3^, who were randomly assigned to co-formulated zidovudine/lamivudine and either abacavir and nevirapine placebo (*n *=* *300), or abacavir placebo and nevirapine (*n *=* *300). After 24 weeks, participants continued to receive the study drugs open-label and were followed up as part of DART. Of those randomized, 32 died before week 96, and 21 were lost to follow-up. A further 107 participants with CD4 counts ≥300 cells/mm^3^ at weeks 48 or 72 after ART initiation were randomized to structured treatment interruption (STI) with up to four repeated cycles of 12 weeks on/off therapy and are excluded. In addition, 7 (4 abacavir, 3 nevirapine) participants had switched to lopinavir-based second-line ART by week 96, leaving 433 participants (236 abacavir, 197 nevirapine), for whom 96.7% of week 48 and week 96 HIV-1 RNA measurements were available.

Viral loads were measured using Roche Amplicor 1.5. Genotypic resistance testing was by standard population sequencing of *pol*, with identification of resistance mutations according to the latest IAS-USA list,^11^ as described previously.^12^ Phenotypic drug resistance was assessed using the Antivirogram assay (v. 2.5.01, Janssen Diagnostics) using a full-length recombinant HIV vector containing patient-derived protease (PR)–reverse transcriptase (RT) sequences up to codon 400 of RT.^13^ Nested RT–PCR was used to amplify a 2.2 kb fragment and the resulting PR–RT-coding sequences were then transfected into CD4+ T lymphocytes (MT4), along with the pGEMT3deltaPRT plasmid. Chimeric viruses containing PR- and RT-coding sequences derived from HIV-1 RNA in plasma were generated through homologous recombination in the MT4 cells, and susceptibilities of chimeric viruses to RT inhibitors were determined by an MT4 cell 3-(4,5-dimethylthiazol-2-yl)-2,5-diphenyltetrazolium bromide-based cell viability assay.^14^ Statistical methods included Student’s *t*-tests and linear regression as appropriate. All *P* values were two-sided. All analyses were performed using STATA v. 14.0 (StataCorp LP, College Station, TX, USA).

### Ethics

Ethics approval both for DART and the NORA sub-study was obtained both in Uganda [Uganda Research Unit on AIDS (UVRI) Science and Ethics Committee] and the UK (Imperial College).

## Results

**Figure 1:**
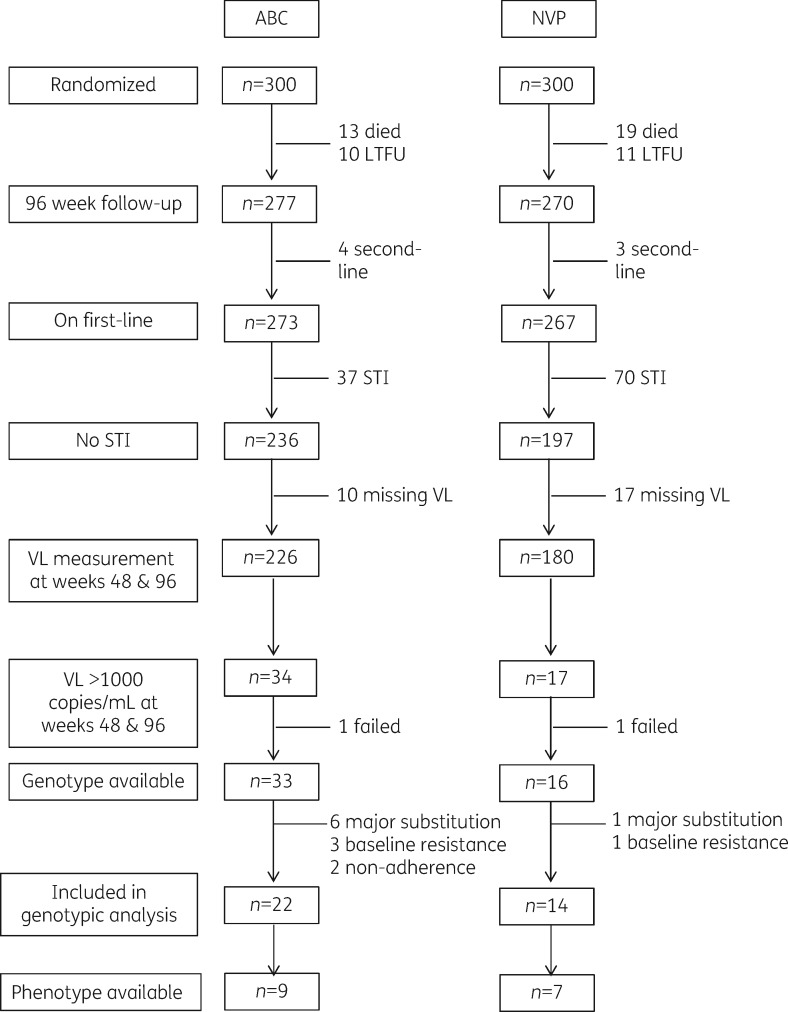
Flow diagram of participants and samples genotyped and phenotyped. ABC, abacavir; NVP, nevirapine; LTFU, lost to follow-up; VL, viral load.

Fifty-one of 433 (12%) participants were identified as having a viral load >1000 copies/mL at both week 48 and week 96 (Figure 1). Genotypic and phenotypic testing was attempted at these timepoints along with a baseline (week 0) sample. Genotyping was successful for 49 pairs of samples. Of these participants, 7 made substitutions to their initial regimen, 4 had detectable resistance mutations prior to starting therapy, and 2 had no major mutations at both weeks 48 and 96, indicating non-adherence; all were excluded from further analyses, leaving 36 individuals with genotype pairs (22 abacavir, 14 nevirapine). Paired phenotypic results from both weeks 48 and 96 were only available in 16 of these 36 participants due to insufficient quantity of sample for phenotyping in the remainder (Figure 1). Twenty-four (67%) of the participants with a genotype were female, with a median (IQR) age at randomization of 37 (30–43) years. The CD4 count at randomization was low, with a median (IQR) value of 44 (17–103) cells/mm^3^.

**Figure 2:**
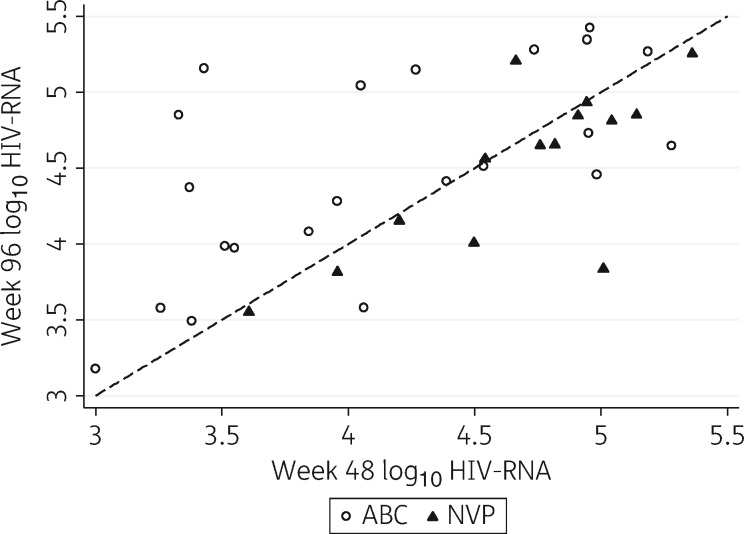
Scatter plot depicting week 48 versus week 96 viral load by treatment group in patients with paired genotypes (*n *=* *36). ABC, abacavir; NVP, nevirapine.

The relationship between an individual’s viral load at weeks 48 and 96 is shown in Figure 2. In participants with paired genotypic data randomized to the nevirapine group (*n *=* *14), mean (SD) viral load was 4.67 (0.49) log_10_ copies/mL at week 48 and 4.52 (0.55) log_10_ copies/mL at week 96 [mean decrease of 0.15 log_10_ copies/mL (95% CI −0.06–0.36)]. In those randomized to abacavir (*n *=* *22), the mean viral load was 4.13 (0.72) log_10_ copies/mL at week 48 and 4.50 (0.66) log_10_ copies/mL at week 96 [mean increase of 0.37 log_10_ copies/mL (95% CI 0.10–0.64)].

Overall, the prevalence of each TAM increased between weeks 48 and 96 in our sample of 36 patients [mean absolute increase 25% (range 19%–31%)]. TAMs at position 215 were particularly frequent (>70% in each arm) followed by D67N (Table 1). In only one case was there a loss of a specific TAM between timepoints (D67N in the nevirapine arm). At 48 weeks, ≥3 TAMs were present in 14% and 23% of nevirapine- and abacavir-treated patients, respectively, and at 96 weeks the proportions were 50% and 73%, respectively. The mean (SE) number of TAMs accumulating between weeks 48 and 96 was 1.50 (0.37) in nevirapine-treated patients and 1.82 (0.26) in abacavir-treated patients. This rate of accumulation was not related to the absolute number of TAMs at week 48 (*P* = 0.38), although numbers in each group were small.

**Table 1:** Prevalence of TAMs and M184V in patients with paired genotypes (*n *=* *36) at weeks 48 and 96, by treatment arm

	ABC (*n* = 22)				NVP (*n* = 14)			
	week 48		week 96		week 48		week 96	
	*n*	%	*n*	%	*n*	%	*n*	%
Mutation								
184V	22	100	22	100	12	86	13	93
41L	2	9	8	36	1	7	4	29
67N	9	41	15	68	5	36	6	43
70R	9	41	14	64	2	14	4	29
210W	0	0	7	32	1	7	5	36
215F	3	14	7	32	0	0	5	36
215Y	3	14	9	41	2	14	5	36
219QE	4	18	11	50	1	7	4	29
No. of TAMs								
0	9	41	3	14	8	57	3	21
1	2	9	1	5	2	14	1	7
2	6	27	2	9	2	14	3	21
3	4	18	4	18	2	14	2	14
≥4	1	5	12	55	0	0	5	36

ABC, abacavir; NVP, nevirapine.

**Figure 3:**
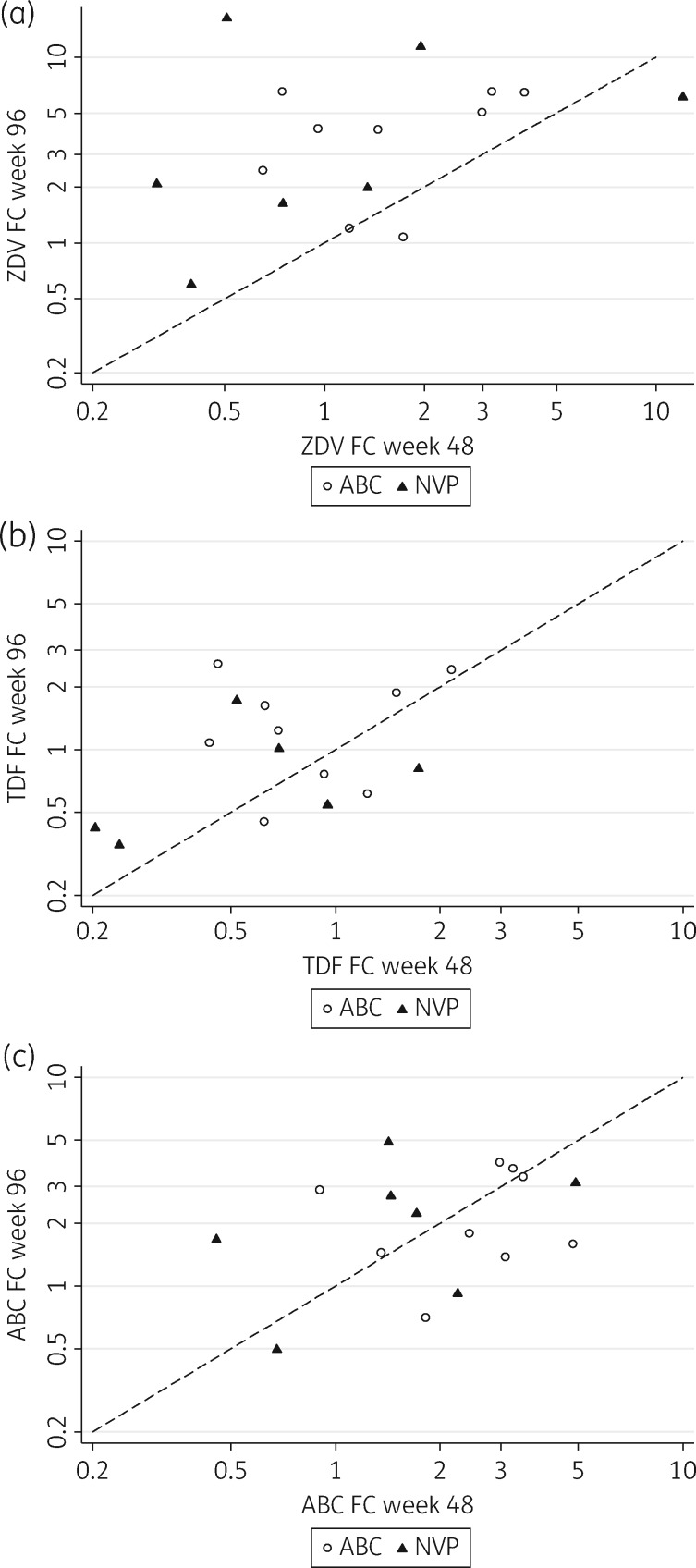
Drug susceptibility of patient-derived virus isolates to abacavir, zidovudine and tenofovir disoproxil fumarate (modelled as FCs relative to a reference isolate) by treatment group in patients with paired phenotypes (*n *=* *16). ABC, abacavir; NVP, nevirapine; TDF, tenofovir disoproxil fumarate; ZDV, zidovudine.

Combining both treatment arms, the zidovudine susceptibility of viruses was reduced between week 48 [geometric mean fold change (FC) 1.3] and week 96 (geometric mean FC 3.4, *P *=* *0.01) (Figure 3a). Similarly, there was a small, non-significant reduction in tenofovir disoproxil fumarate susceptibility over time (FC 0.7 and 1.0, respectively, *P *=* *0.18) (Figure 3b) with most patients remaining fully susceptible. However, there were no observed changes in susceptibility to abacavir (FC 1.9 and 1.9, respectively, *P *=* *0.96) between these timepoints (Figure 3c).

Lamivudine resistance (M184V) was present in all patients with viral failure at 96 weeks except one participant on the nevirapine arm (Table 1), and had emerged by week 48 in all but one patient on the nevirapine arm. Other NRTI-associated mutations were present only in participants on the abacavir arm, and had emerged by week 48: one individual acquired the multinucleoside resistance 69 insertion, one acquired K65R and two acquired Y115F (Table S1, available as Supplementary data at *JAC* Online). The abacavir-selected mutation L74V mutation was not observed in any participant (Table S1).

The connection domain mutation N348I conferring resistance to zidovudine^15^^,^^16^ was acquired in 4/14 (28.6%) of nevirapine-treated patients and in 6/22 (27.3%) of abacavir-treated patients.

Major NNRTI mutations occurred in 12/14 participants treated with nevirapine by week 48. In those who were already resistant by week 48, only one participant acquired a new mutation by week 96 (V106A at week 48, K101E + G190A at week 96), and both participants without NNRTI mutations at week 48 went on to develop G190A by week 96.

## Discussion

NNRTI-based cART appears to be associated with rapid accumulation of TAMs in those with virological failure (1.50 TAMs per year for zidovudine/lamivudine/nevirapine-treated patients) in this resource-limited setting where routine viral load monitoring is not performed. We observed that abacavir/zidovudine/lamivudine also resulted in rapid TAM accumulation (1.82 TAMs per year), although WHO guidelines do not currently recommend triple nucleoside-based cART for first-line cART. Our estimate of TAM accumulation for NNRTI-based ART is higher than reported previously in South African adults^17^ (0.84 per year), and in Zambian children^18^ (0.5 per year). Both those studies involved significant stavudine use, as opposed to zidovudine. Stavudine is known to select not only for TAM mutational pathways, but also selects for K65R and this could partly explain the discrepancy with our data.

Over half of all patients with viral failure by week 48 in this study had ≥3 TAMs in total by week 96. Accumulated TAMs were associated with reduced phenotypic drug susceptibility amongst a smaller sample of patients, with zidovudine susceptibility significantly falling between weeks 48 and 96, and a small non-significant decrease observed for tenofovir.

We know from previous studies that boosted PI-based first-line ART is protective against the development of NRTI mutations, in comparison with NNRTI-based first-line therapy.^19–21^ This is important to bear in mind as first-line treatment strategies in sub-Saharan Africa may need to be changed in the coming years due to the rising prevalence of transmitted NNRTI drug resistance.^22^ It will be of interest to know whether the use of integrase inhibitors is also protective against NRTI resistance in such settings.

### Limitations

This study was limited by a relatively small number of available phenotype pairs and the conclusions are largely drawn from genotypic information. Furthermore the data are derived from nevirapine-treated patients and efavirenz is now the most widely used NNRTI in resource-limited settings.^23^

Despite its associated toxicity, zidovudine has emerged as an important component of cART regimens in resource-limited settings; moreover, dose reduction reduces incidence of moderate to severe anaemia.^24^ WHO recommends the use of zidovudine in second-line regimens following failure of first-line regimens containing tenofovir. This presents a possible problem where patients have accumulated TAMs prior to tenofovir replacing zidovudine during first-line cART, though two large studies have not shown a detrimental effect of TAMs on boosted PI-based second-line therapy.^25^^,^^26^

Transmission of NRTI drug resistance compromises subsequent response to first-line therapies,^27^^,^^28^ and therefore limitation of accumulated TAMs should be a public health priority as cART is scaled up. It is also appropriate to limit multidrug resistance as this may allow for recycling of drugs in the future as the epidemic matures.

Effective viral load monitoring has been identified as a priority area^29^ and could avoid prolonged viral failure with accumulation of TAMs.^30^ In such individuals with virological failure, adherence counselling with possible switch to second-line therapy should be undertaken, thus avoiding ongoing first-line failure and accumulation of drug resistance.^8^

## Supplementary Material

Supplementary Data
