# Supplementary material for: Rapid accumulation of HIV-1 thymidine analogue mutations and phenotypic impact following prolonged viral failure on zidovudine-based first-line ART in sub-Saharan Africa
Source: J Antimicrob Chemother. 2017 Feb 4;72(5):1450–5. doi: 10.1093/jac/dkw583 (PMC5400089; doi:10.1093/jac/dkw583)
Supplement: Supplementary Data [file dkw583_Supp.docx]

**Supplementary data**

**Supplementary Table 1**: Individual RT genotypic data by week from ART initiation

| Patient | Week | RT mutations present |
| --- | --- | --- |
| NVP |  |  |
| 1 | 48 | 106A 184V |
|  | 96 | 101E 184V 190A |
| 2 | 48 | 181C 184V 210W 215Y 41L |
|  | 96 | 181C 184V 210W 215Y 41L |
| 3 | 48 | 184V 190A |
|  | 96 | 184V 190A 210W 215Y |
| 4 | 48 | 101E 184V 190A |
|  | 96 | 101E 184V 190A 215F 219Q 67N 70R |
| 5 | 48 | 181C 184V |
|  | 96 | 181C 184V 210W 215F 41L 67N |
| 6 | 48 | 106A |
|  | 96 | 184V 190A 210W 215Y |
| 7 | 48 | 181C 181V 184V 67N |
|  | 96 | 181V 184V 215F |
| 8 | 48 | 101E 184V 190A |
|  | 96 | 101E 184V 190A 215Y 41L |
| 9 | 48 | 184V 190A |
|  | 96 | 184V 190A |
| 10 | 48 | 103N 184V 67N |
|  | 96 | 103N 184V 219Q 67N 70R |
| 11 | 48 | 184V 219Q 67N 70R |
|  | 96 | 184V 190A 215F 219E 67N 70R |
| 12 | 48 |  |
|  | 96 | 190A |
| 13 | 48 | 184V 190S 215Y 67N |
|  | 96 | 184V 190S 210W 215Y 41L 67N |
| 14 | 48 | 103N 184V 67N 70R |
|  | 96 | 103N 184V 215F 219E 67N 70R |
| ABC |  |  |
| 15 | 48 | 184V 219Q 67N 70R |
|  | 96 | 184V 215F 219E 41L 67N 70R |
| 16 | 48 | 184V 70R |
|  | 96 | 184V 215F 219E 67N 70R |
| 17 | 48 | 184V |
|  | 96 | 184V |
| 18 | 48 | 184V |
|  | 96 | 184V 215Y |
| 19 | 48 | 184V 67N 70R |
|  | 96 | 184V 219E 67N 70R |
| 20 | 48 | 184V |
|  | 96 | 184V 67N 70R |
| 21 | 48 | 184V 65R |
|  | 96 | 184V 65R |
| 22 | 48 | 184V 219E 67N 70R |
|  | 96 | 184V 215F 219E 67N 70R |
| 23 | 48 | 184V |
|  | 96 | 184V 219Q 67N 70R |
| 24 | 48 | 184V |
|  | 96 | 115F 184V |
| 25 | 48 | 184V 67N 70R |
|  | 96 | 184V 210W 215Y 41L 67N 70R |
| 26 | 48 | 184V |
|  | 96 | 184V 210W 215Y 225H 41L |
| 27 | 48 | 184V 67N 70R |
|  | 96 | 184V 215F 219E 67N 70R |
| 28 | 48 | 184V 215Y 67N 70R |
|  | 96 | 184V 210W 215Y 219E 41L 67N 70R |
| 29 | 48 | 184V 219E 70R |
|  | 96 | 184V 215F 219E 41L 70R |
| 30 | 48 | 184V |
|  | 96 | 184V 215Y 219E 67N 70R |
| 31 | 48 | 184V 215F 41L 67N |
|  | 96 | 184V 210W 215Y 41L 67N |
| 32 | 48 | 115F 184V 215F 69SIns |
|  | 96 | 115F 184V 210W 215F 215Y 69SIns |
| 33 | 48 | 184V 215F 219E 67N 70R |
|  | 96 | 184V 215F 219E 67N 70R |
| 34 | 48 | 184V |
|  | 96 | 184V 219Q 67N 70R |
| 35 | 48 | 115F 184V 215Y 67N |
|  | 96 | 184V 210W 215Y 41L 67N 70R |
| 36 | 48 | 184V 215Y 41L |
|  | 96 | 184V 210W 215Y 41L 67N |
